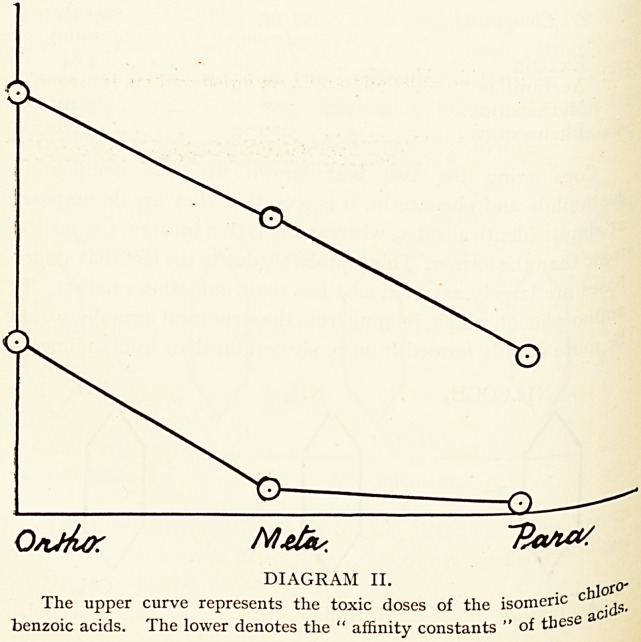# The Relationship between Toxicity and Chemical Reactivity in Certain Benzene Derivatives

**Published:** 1912-09

**Authors:** Oliver C. M. Davis

**Affiliations:** Lecturer on Materia Medica in the University of Bristol


### THE RELATIONSHIP BETWEEN TOXICITY AND CHEMICAL REACTIVITY IN CERTAIN BENZENE DERIVATIVES.

BY

OLIVER C. M. DAVIS, D.Sc.,

*Lecturer on Materia Medica in the University of Bristol.*

---

IN determining the pharmacological value of an organic compound, information can best be obtained by direct experiments on living animals, and all other means of investigation must be regarded as of secondary importance.

In many cases it appears that this may be largely due to the fact that the nature of the decomposition which a compound undergoes within the organism may differ essentially from its decomposition *in vitro*, and this makes it difficult to predict the action of any new drug until its products of decomposition *in vivo* have been ascertained. With the knowledge at present available it is quite obvious that it cannot always be possible to foretell the physiological action of a drug from its chemical properties and stability; but in the present paper an attempt is made to show that in certain cases there is a direct and apparently quantitative relationship between chemical constitution and toxicity. In order to study this relationship, it is necessary to select some definite series of organic compounds which (*a*) can be prepared in a state of great purity, (*b*) react in an identical manner with certain re-agents, and (*c*) possess a well-marked and similar physiological action.

Unfortunately, there is not a very large number of chemical series available for this purpose, but certain aniline derivatives have been investigated from both chemical and pharmacological standpoints, and the comparative results are of special interest to the student of pharmaco-dynamics.

For a considerable number of years certain of the "anilides" have found an application in practical medicine, being used as antipyretics. They are the "acyl" derivatives of aniline and its congeners, and their activity is due primarily to the fact that they are slowly hydrolysed within the organism, giving rise to the amine and acid from which they are derived according to the general equation :—

$$R.NH.Ac + H.OH \rightleftharpoons R.NH_2 + AcOH.$$

Small quantities of the free base are thus gradually introduced into the system, and a medicinal effect produced side by side with a very greatly diminished toxicity compared with that of the parent amine. One of the first members of the series to be used for medicinal purposes was the acetyl derivative of aniline or acetanilide, commonly known as antifebrin, which has been largely employed under this name, and also as a constituent of proprietary headache powders.

Within the alimentary system this compound is hydrolysed with liberation of free aniline and acetic acid or their salts.

Experiments have been made by physiologists on the toxicity of numerous anilides, and the following conclusions have been drawn ([1]) :—

That the toxicity of these compounds gradually diminishes as the length of the carbon chain in the "acyl" group increases; thus formanilide, unlike its immediate homologue acetanilide, is far too toxic for internal administration, and in the aromatic series benzanilide is practically without physiological action. Some few years ago the rates of decomposition of a considerable number of anilides were investigated, hydrolysis being brought about by boiling with standard sodium hydroxide, according to the equation ([2]) :—

$$R.NHAc. + NaOH \rightarrow RNH_2 + AcONa.$$

The following results were obtained, " K " being a constant denoting the average velocity of decomposition :—

| *Compound.* | *Time for* 50 % *decomp.* | *K.* |
|---|---|---|
| Formanilide .. .. .. | 0.14 hrs. .. .. | 87.1 |
| Acetanilide .. .. .. | 3.79 " .. .. | 3.20 |
| Propionanilide .. .. .. | 6.30 " .. .. | 1.93 |
| N. Butyranilide.. .. .. | 13.0 " .. .. | 0.934 |
| N. Valeranilide .. .. .. | 18.5 " .. .. | 0.656 |
| Benzanilide .. .. .. | Not affected. | |

On the assumption that the physiological action of this series is primarily due to the liberation of the free amine, it is quite obvious from these results that formanilide would prove far more toxic than acetanilide, since the constant indicates that the former compound is decomposed approximately twenty-seven times as quickly as the latter ; the results also lead us to suppose that toxicity would diminish with increasing molecular weight of the acid from which the compounds are formed.

During the last twenty years a considerable amount of research has been carried out on the rates of formation and decomposition of numerous organic compounds ; the results obtained are of great interest, but are incomplete in themselves, as in all probability two distinct factors are involved, which determine the reactivity of organic compounds, namely their " chemical affinity " or power to react, and also the " frictional resistance " which they offer to any given reaction. It is therefore obvious that rates of reaction may be influenced by one or both of these factors, and thus a determination of velocity constants only does not give us a direct measurement of chemical affinity. There are two recognised methods for measuring this property, one being to determine the electro motive forces of the substances under investigation, and the other to find the position of equilibrium in a balanced action, and calculate the affinity from recognised formulæ in which the equilibrium constant is introduced.

Although from a theoretical standpoint all chemical reactions are reversible if suitable conditions are chosen, yet the number of definite cases where distinct equilibria can be attained and accurately measured is not great. A series of such reactions has recently been investigated,[3] the interaction of formic acid with aniline bases in pyridine-water solution having been traced from both sides of the equation :—

$$R.NH_2 + HCOOH \rightleftarrows R.NH.HCO + H_2O.$$

A large number of bases were experimented with, and the equilibrium constants calculated from the formula—

$$K_W = \frac{C_1}{C_2 \times C_3}, \text{ where}$$

$K_W$ = equilibrium constant, and $C_1$, $C_2$ and $C_3$ the respective concentrations of anilide, amine and acid at the position of equilibrium.

Unfortunately, at the present time only a few of the bases investigated have had their relative toxicities determined. Diagram I was obtained by plotting both the reciprocals of $K_W$ and the toxic doses per kilogramme body-weight of the toluidines[4] on equidistant ordinates to empirically represent the ortho, meta, and para-orientations. It is well shown that toxicity is approximately directly proportional to chemical affinity, since this property varies with the equilibrium constant and toxicity is inversely proportional to toxic dose.

In this connection two well-marked cases have been thoroughly worked out, showing the application of theory to practice.

It has been found by Breinl and Nierenstein[5] that whereas p. amino-phenyl-arsenic acid, the sodium salt of which is known as atoxyl, acts promptly on trypanosomes, the meta-compound has a less effect, and the ortho-compound no effect at all. A similar regularity was noticed by the same observers[6] in the case of the corresponding antimony compounds.

DIAGRAM I.

The upper curve represents the toxic doses of the isomeric toluidines, the lower curve denotes the reciprocals of the equilibrium constants of the toluidines reacting with formic acid (N / I solutions in pyridine and water).

The relationship between the compounds is shown by the following formulæ :—

*Arsenic Compounds.*

*Ortho*—No effect. *Meta*—Slight effect. *Para*—Effective.

*Antimony Compounds.*

*Ortho*—No effect. *Meta*—Slight effect. *Para*—Effective.

These investigators are of the opinion that these differences are due to the different reactivities of the amino groups towards the proteins, as they were able to show that the action of the drugs on the parasites only takes place after they have combined with protein. [7]

Some further interesting work on the importance of the amino group (—$NH_2$) as an "anchoring group" have also been carried out with "atoxyl" by Nierenstein, Breinl and others, who showed that by replacing —$NH_2$ by —OH, —$N(CH_3)_2$ and —H, the compound loses its action on trypanosomes [8] both *in vitro* and *in vivo*. This is expressed best by the aid of graphic formulæ.

Effective. Not effective.

Quite recently Ehrlich's co-worker, Bertheim, has shown that the well-known drug "606" becomes ineffective when —$NH_2$ is replaced by —$N(CH_3)_2$. [9]

Effective. Ineffective.

There are, however, certain cases where chemical reactivity and toxicity have both been determined, with results which at first appear to be at variance with the previous observations.

For instance, on determining the rates of hydrolysis of the four compounds exalgin, acetanilide, methacetin and phenacetin, the following values were obtained :—[10]

| *Compound.* | *Time for 50 per cent. decomp.* | *K. (average velocity constant).* |
|---|---|---|
| Exalgin .. .. .. .. .. | 3.16 hrs. .. .. | 3.84 |
| Acetanilide .. .. .. | 3.79 ,, .. .. | 3.20 |
| Methacetin.. .. .. .. | 3.57 ,, .. .. | 3.40 |
| Phenacetin.. .. .. .. | 3.70 ,, .. .. | 3.28 |

Considering the two best known of these compounds, acetanilide and phenacetin, it is seen that they are decomposed at almost identical rates, whereas the latter substance is far less toxic than the former. This is probably due to the fact that aniline bases are largely excreted as a less toxic oxidation product. P. amino-phenol, which, judging from the structural formulæ, would be more readily formed from p. phenetidin than from aniline.

Acetanilide. Aniline. p. Amino-phenol.

Phenacetin. p. Phenetidin. p. Amino-phenol.

An interesting relationship is also noticed when we compare the " affinity constants " of the chlor-benzoic acids [11] with their toxicities, and find the two factors inversely proportional to each other. This is well shown in Diagram II, where the toxic doses per kilogramme body-weight are plotted on the same ordinates as the affinity constants, the resultant curves running in the same direction.

DIAGRAM II.

The upper curve represents the toxic doses of the isomeric chloro-benzoic acids. The lower denotes the " affinity constants " of these acids.

The question naturally arises why the toxicities should, in the case of the toluidines, be proportional to their chemical affinities, and with respect to the chlor-benzoic acids inversely proportional to these constants. When we consider each case separately, however, an explanation can be offered, since in both instances it may safely be stated that toxicity must depend on three factors, namely :—

The affinity of the compound for protoplasm, *i.e.* the reactivity of the "anchoring group" (in the case of the toluidines $—NH_2$).

The vigour with which the "anchored" main portion of the molecule (in the case of the toluidines $C_6H_4CH_3—$)

(*a*) Behaves towards protoplasm.

(*b*) Gives rise to excretion products.

Thus we may write a hypothetical equation :—

$$\text{Drug} + \text{cell} \rightleftharpoons \text{cell-drug.}$$

During the next stage two reactions may be taking place side by side, one producing a physiological effect, and the other giving rise to excretion products, and we can assume that where there is a great affinity between cell and drug the pharmacological action will be correspondingly great, and will be diminished by any factor which increases the rate of excretion of the drug.

Now it has been observed that the isomeric chlor-toluenes ($C_6H_4Cl.CH_3$) are first oxidised within the body to chlor-benzoic acids, ($C_6H_4Cl.COOH$), and then excreted as the corresponding chlor-hippuric-acids, which are condensation products of chlor-benzoic acids with glycine. It has also been shown that "ortho" chlor-toluene is the least toxic and "para" chlor-toluene the most toxic of the three isomers [1 2].

The chlorinated benzoic acids, when administered as such, would doubtless also be excreted as chlor-hippuric acids, and we should expect that isomer with the greatest "affinity" for glycine, *i.e.* the one with the greatest affinity constant, to be most readily excreted, therefore less toxic than the other isomers.

It is, of course, quite probable that excretion may take place without the formation of a "cell-drug" compound, but this does not materially alter the views herein suggested. It therefore seems desirable to introduce some new terms into the literature on the subject, and to express the relationship by means of an equation which would of necessity only be of a qualitative nature. Thus the term "physiological affinity" might be used to express the readiness with which a chemical compound reacts with protoplasm, and this in many cases would doubtless be closely related to the "chemical affinity" of the compound in question. The ease with which the same compound gives rise to excretion products might also be denoted by the magnitude of a hypothetical "excretion constant," and a new expression would be necessary :—

$$\text{Toxicity constant} = \frac{\text{Physiological affinity.}}{\text{Excretion constant.}}$$

If we now examine a few special cases from this point of view a new light is thrown on certain observed facts. It is not unreasonable to suppose that in the case of the toluidines the "ortho" compound is least toxic, because it possesses the lowest "physiological affinity," and the relative ease with which it is excreted does not counteract this factor.

The same remarks would apply to the arsenic and antimony compounds investigated by Breinl and Nierenstein (*loc. cit.*). With regard to the hydroxy-benzoic acids, it is generally stated that the "ortho" compound (salicylic acid) is the only isomer which is physiologically active. The "affinity constants" of these acids are :—[13]

| | |
|---|---|
| Ortho hydroxy-benzoic acid | 0.102. |
| Meta ,, ,, ,, | 0.00867. |
| Para ,, ,, ,, | 0.00286. |

In all probability the hydroxyl group serves as the chief "anchoring group," and we might expect that isomer having the highest chemical affinity to possess the greatest physiological activity, provided the "excretion constant" does not upset this relationship, as is the case of the chlor-benzoic acids. It should here be mentioned that according to Hildebrandt[14] salicylic acid is not eliminated in the same way as its isomers, and this may have an influence on its toxicity.

I would emphasise the fact that the ideas brought forward in this paper are merely speculative, but it is hoped that further work may throw more light on an important and fascinating problem.

My thanks are due to the Government Research Committee of the Royal Society for a grant to defray the expenses of some of the chemical work herein mentioned.

### REFERENCES.

1 H. Hildebrandt, *Neuere Arzneimittel*, 1907, p. 13.

2 O. C. M. Davis, *Tr. Chem. Soc.*, 1909, p. 1397.

3 O. C. M. Davis, *Ztschr. Physikal. Chem.*, 1911, lxxviii. 353–368.

4 Fränkel, *Arzneimittel Synthese*, second edition, p. 243.

5 Breinl and Nierenstein, *Ann. Trop. M. and Parasitol.*, 1909, iii. 395.

6 *Ibid.*, p. 365.

7 Breinl and Nierenstein, *Ztschr. f. Immunitätsforsch., etc.*, 1909, i. 620; Nierenstein, *Ann. Trop. M. and Parasitol.*, 1908, ii. 249, 323.

8 Moore, Nierenstein and Todd, *Ann. Trop. M. and Parasitol.*, 1908, ii. 271; Nierenstein, *Ibid.*, p. 249; Breinl and Nierenstein, *Ibid.*, 1909, iii. 395.

9 Bertheim, *Ber. d. Deutchen Chem. Gesellsch.*, 1912, xlv. 2136.

10 O. C. M. Davis, *loc. cit.*

11 W. Ostwald, *Ztschr. Physikal. Chem.*, 1889, iii. 255, 256.

12 H. Hildebrandt, *Beitr. z. Chem. Pathol.*, 1902, Bd. 3.

13 W. Ostwald, *loc. cit.*, p. 247.

14 Hildebrandt, *Ztschr. Physiol. Chem.*, 1904, Bd. 43.

---

### RAYNAUD'S DISEASE.

BY

DAVID A. ALEXANDER, M.B., Ch.B.Vict.

## Figures and Tables

**DIAGRAM I. f1:**
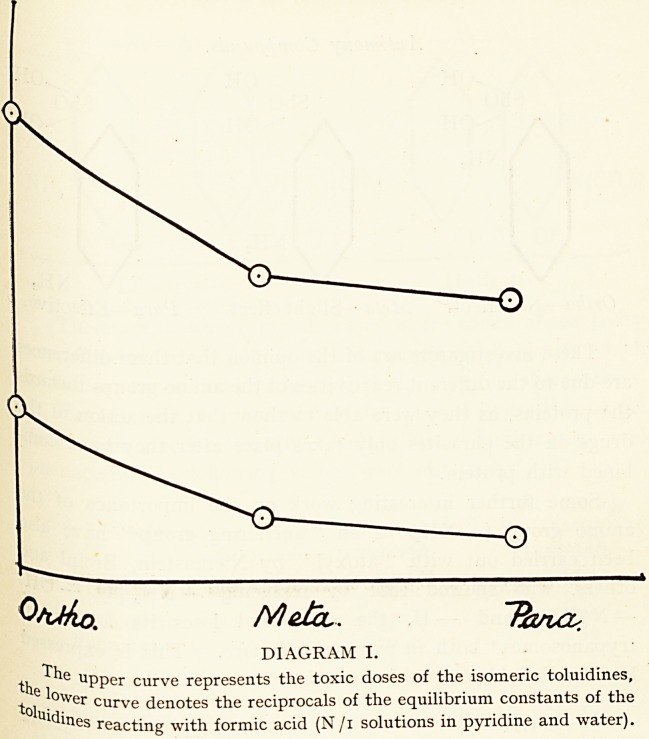


**Figure f2:**
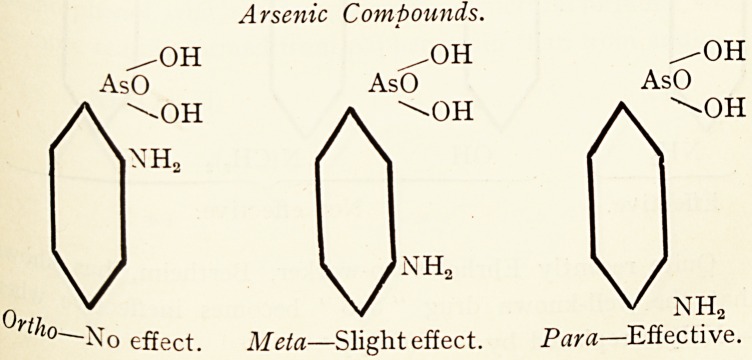


**Figure f3:**
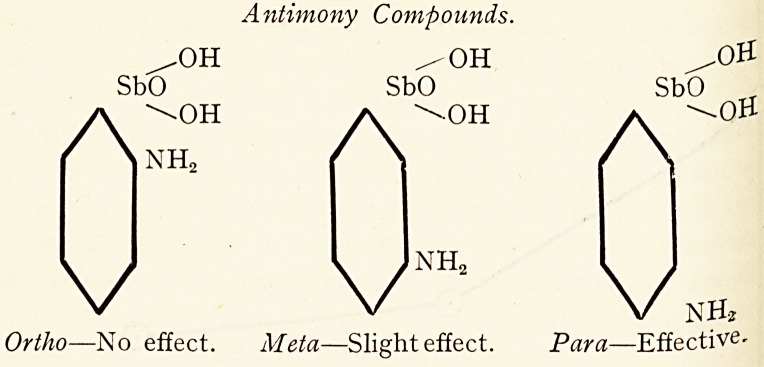


**Figure f4:**
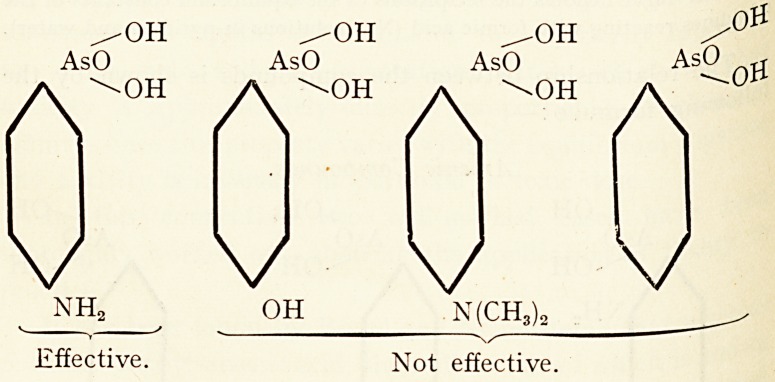


**Figure f5:**
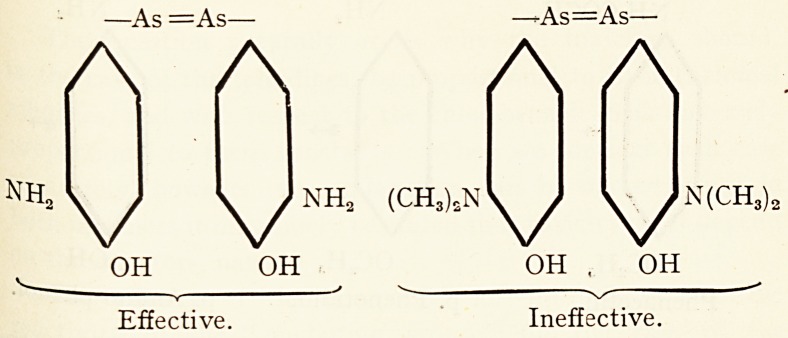


**Figure f6:**
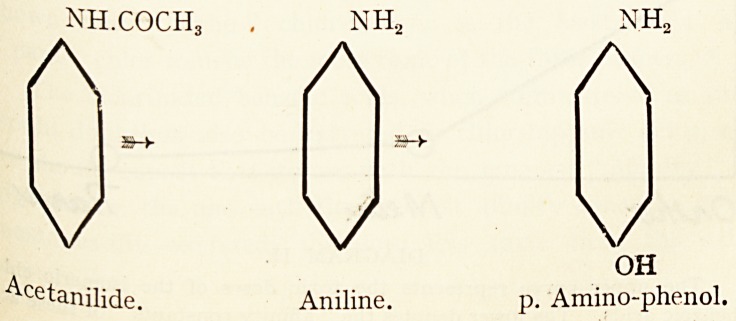


**Figure f7:**
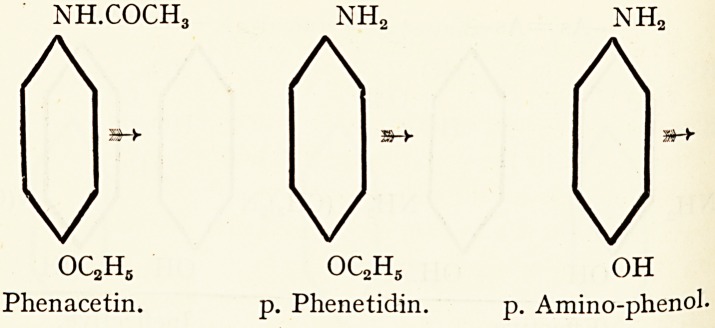


**DIAGRAM II. f8:**